# Assessment of dentists' knowledge, attitude and practice regarding artificial intelligence in periodontics

**DOI:** 10.6026/973206300222712

**Published:** 2026-05-31

**Authors:** S.H Bharathi, B Mohammed Ismail, K Hanumantha Kumar, K Sadananda, B Radhika, Shweta Halemani

**Affiliations:** 1 Department of periodontics, Government Dental College and Research Institute, Ballari Medical College and Research Centre, Ballari, Karnataka, India

**Keywords:** Artificial intelligence (AI), periodontics, knowledge, attitude, practice, dental education, digital dentistry

## Abstract

Artificial intelligence is rapidly transforming periodontal diagnosis, treatment planning and patient management; however, its adoption among dental professionals remains limited. Therefore, it is of interest to evaluate the knowledge, attitude and practice of dentists regarding artificial intelligence in periodontics among 800 participants recruited through stratified random sampling from January to June 2024. Data were collected using a structured, pre-validated questionnaire and analyzed using descriptive statistics, chi-square test and ANOVA. Although 75% of participants were aware of artificial intelligence and 80% expressed willingness to adopt it, only 25% reported its actual use in clinical practice, with concerns regarding data privacy and professional displacement noted in 40% of respondents. A significant gap exists between positive perception and clinical implementation, highlighting the need for targeted education, ethical guidelines and improved accessibility to artificial intelligence technologies in periodontics.

## Background:

Artificial intelligence (AI) has turned out to be a groundbreaking technology in practically all spheres of healthcare and provides advanced computing capabilities to improve diagnosis, treatment and clinical decision-making [1]. In the wider context of medical innovation, dentistry has been gradually adopting digital technologies and the latest addition to the field of continuous evolution is the artificial intelligence. Implementation of machine learning algorithms, neural networks and deep learning architectures in dental practice has shown important potentials in supplementing the clinical functions of the traditional method of diagnosis and treatment in a way that was previously not possible [2, 3]. The dental specialty that would be the most impacted by artificial intelligence applications is periodontics that deals with the prevention, diagnosis and management of diseases that impact the tooth-supporting structures. Periodontal diseases form a critical health problem globally, having a prevalence of about 50 percent among the adults in the world and is one of the major causes of tooth loss in adults [4]. The multifactorial nature of periodontal pathology, combined with the radiographic and clinical subtleties of its initial forms, produces the opportunity of conditions under which diagnostic aids based on artificial intelligence can make a significant contribution to the detection rates and therapeutic outcomes. Some of the existing applications include automated radiographic interpretation in alveolar bone loss measurements, disease progression predictions, computerized periodontal charting programs and algorithmic treatment planning software [5, 6]. Although the potential of these technologies proved demonstrative, the transfer of the artificial intelligence of the research setting to the everyday periodontal practice has been particularly slow.

Various challenges have been listed to limit adoption, which include practitioner education levels in artificial intelligence potential and constraints, distrust over the credibility and accuracy of the algorithm outputs, fear over the ethical issues like patient information security and professional autonomy and pragmatic issues associated with costs and technology infrastructure [7, 8]. The effective adoption of artificial intelligence in the field of clinical periodontics depends essentially on the openness and readiness of the dental professionals who are the key to the speed and scope of its adoption through the prism of their knowledge and perceptions as well as behavioral participation in such technologies. Learning the knowledge, attitude and practice patterns of dental professionals in artificial intelligence is thus critical in determining the areas of shortcomings, the practitioner concerns and establishing specific strategies to aid the adoption. Attitudes towards artificial intelligence of healthcare workers of different medical specialties have been studied before, but no through evaluations of attitudes specifically oriented at the periodontic setting have been conducted [9, 10]. Therefore, it is of interest to critically assess the knowledge, attitude and practice of dentists in the application of artificial intelligence in periodontics with the view of establishing action-could knowledge to inform education programs and enable responsible adoption of artificial intelligence technologies in periodontal care.

## Materials and Methods:

## Design and ethical concerns of the study:

It is a cross-sectional observational study, but the participant population will include several dental institutions and individual dental clinics based on their experience in the specified period (January to June 2024).

## Population and sampling of the study:

The population was a sample of licensed dental practitioners that were involved in active clinical practice or postgraduate training. The sample size was found to be 800 subjects; this is due to the expected response rate of 50 participants, confidence level of 95 and a margin of error of 5, which was adjusted because of non-response. The stratified random sampling method was used to select the participants to facilitate an adequate representation of the three pre-defined professional strata, such as general dental practitioners, specialist periodontists and postgraduate dental students. The stratification aimed at representation of possible divergent views at various levels of specialization and clinical experiences. The inclusion criteria included registered dental practitioners who had one year of clinical experience or those who are enrolled in accredited postgraduate dental programs. The exclusion criteria were the practitioners who were retired, on prolonged leave of clinical practice and those who refused to give informed consent.

## Data collection instrument:

The data were gathered with the help of a self-administered questionnaire based on the extensive research of the available literature on artificial intelligence in healthcare and developed with references to the pilot study groups of 30 dentists who did not participate in the main analysis. The questionnaire was evaluated with regard to content validity by a team of five experts that included periodontists, dental informaticians and biostatisticians. Cronbach alpha coefficient was used to assess internal consistency and it had a value of 0.84 which is acceptable reliability.

## The questionnaire had four parts that had 32 items altogether:

Section I - Demographics (8 items): Age, gender, professional highest qualification, clinical experience years, kind of practice environment, place of residence, availability of digital technologies and previous exposure to artificial intelligence education. Section II - Knowledge (10 items): The items that measure knowledge of the basic concepts of artificial intelligence, awareness of particular application of artificial intelligence in periodontics, knowledge of machine learning and deep learning concepts, familiarity with commercially available artificial intelligence tools that can be used in periodontics and the knowledge of any limitations and possible error of artificial intelligence systems are measured. Section III - Attitude (8 items): Items that measure the perception of artificial intelligence as reliable and accurate, willing to integrate artificial intelligence into clinical workflows, perceptions about whether artificial intelligence could affect professional autonomy, concerns about the ethical consequences of the use of artificial intelligence (data privacy, informed consent) and perceptions of artificial intelligence as part of dental education curriculum. Section IV - Practice (6 items): It includes questions on the present use of artificial intelligence tools or software in clinical practice, how often it was used, what was used, how it influenced clinical outcomes, what were the barriers to its use and what plans they had in the future about the use of artificial intelligence. The survey was administered online through email and messaging service. Two reminder messages were also dispatched after two weeks so as to maximize the response rates.

## Statistical analysis:

All of the data gathered were coded, inputted and analyzed with the Statistical Package of Social Sciences (SPSS, version 26.0; IBM Corp., Armonk, NY). All the variables were calculated using descriptive statistics such as frequencies, percentages, means and standard deviations. Demographic variables were compared to chi-square tests categorical variables and assessed the association between demographic characteristics and knowledge, attitude and scores of practice. ANOVA was used to test the mean scores of the professional categories with the use of the Tukey post hoc analysis in the cases of the significant difference identification. All analyses had a level of statistical significance of p < 0.05.

## Results:

Of the 800 questionnaires distributed, all were completed and returned, yielding a response rate of 100%. The study sample comprised 400 general dental practitioners (50.0%), 250 periodontists (31.3%) and 150 postgraduate students (18.7%). The mean age of participants was 35.2 ± 8.6 years. Female respondents constituted 60.0% of the sample, while males represented 40.0%. The mean duration of clinical experience was 9.4 ± 6.8 years. The knowledge assessment revealed varying levels of awareness across different domains of artificial intelligence (Table 1). A substantial majority of respondents (75.0%) demonstrated familiarity with basic artificial intelligence concepts, including general definitions and broad healthcare applications. Knowledge of specific artificial intelligence applications relevant to periodontics was reported by 65.0% of participants. However, awareness of commercially available artificial intelligence-based tools specifically designed for periodontal use was considerably lower, with only 40.0% of respondents indicating familiarity with such products. Understanding of the inherent limitations and potential sources of error in artificial intelligence systems was reported by 60.0% of participants. Significant differences in overall knowledge scores were observed across professional categories (F = 14.82, p < 0.001), with periodontists demonstrating the highest mean knowledge scores, followed by postgraduate students and general practitioners. Participants under 35 years of age exhibited significantly higher knowledge scores compared to older cohorts (p = 0.003). Assessment of attitudes revealed predominantly positive perceptions toward artificial intelligence in periodontics (Table 2). Seventy percent of respondents expressed confidence in the reliability of artificial intelligence for dental applications and a notable 80.0% indicated willingness to adopt artificial intelligence tools if made available. Despite these favorable attitudes, only 25.0% of respondents reported current utilization of any form of artificial intelligence in their clinical practice. Ethical concerns were identified by 40.0% of participants, with data privacy, patient confidentiality and potential professional displacement cited as the most frequently reported apprehensions. Among the 600 respondents who were not currently utilizing artificial intelligence in their practice, the most frequently cited barriers included insufficient training and education (68.3%), high cost of artificial intelligence technologies (54.2%), lack of institutional support and infrastructure (47.5%), concerns about the accuracy and reliability of artificial intelligence outputs (38.3%) and ethical and medicolegal uncertainties (32.5%). Chi-square analysis revealed significant associations between professional qualification and practice adoption (χ^2^ = 22.46, p < 0.001), with periodontists demonstrating the highest rate of artificial intelligence utilization (36.0%), followed by postgraduate students (24.0%) and general practitioners (18.5%) (Table 3).

## Discussion:

The research results of the current study present a detailed description of the existing knowledge, attitude and practice of dental practitioners in the field of artificial intelligence use in periodontics, which demonstrates a significant gap between positive views and the practice. This knowledge-attitude-practice gap turns out to be the key revealing of the research and has strong implications on the strategy of education, the development of technology and the development of policies in the dental profession. The fact that three-quarters of the respondents reported being familiar with the basic concepts of artificial intelligence indicates the growing ubiquity of artificial intelligence discussion in the mainstream professional consciousness, probably due to the high volume of media coverage, the emergence of clinical literature and the increased availability of artificial intelligence products to consumers [11, 12]. Nonetheless, the much smaller awareness of periodontics-specific artificial intelligence tools, with only 40 percent of the respondents reporting that, does indicate that general conceptual familiarity has not been converted into domain-specific practical knowledge. This observation agrees with other surveys undertaken in the other medical specialties where practitioners had sufficient theoretical knowledge of the principles of artificial intelligence but had little knowledge of the specific application of the technology in the specialty [13, 14]. The gap between basic and applied knowledge highlights the importance of education programs that go beyond the introductory ideas of artificial intelligence to offer direct exposure of practical applications of tools and workflows relevant in clinical environments.

The attitudinal profile which is predominantly positive that was revealed in this research with high rates of confidence in the reliability of the artificial intelligence and strong intention to use the artificial intelligence technologies indicates that attitudinal resistance is not the main obstacle to implementation. This result is consistent with the growing international evidence that dental professionals tend to be open to technological innovation and that they can appreciate the possible benefit of artificial intelligence to improve the accuracy of the diagnosis and clinical efficiency [15, 16]. The willingness to adopt rate of 80 percent recorded in the current study is quite promising given that it shows that there is a significant latent demand of artificial intelligence solutions which can be tapped using proper educational and infrastructural facilitation. The comparison of expressed willingness and actual practice adoption being starkly different is probably the most far-reaching result of this inquiry. It is noteworthy that the respondents rate the integration of artificial intelligence into their clinical practice as only 25 percent of the respondents mentioned that they had used it and had such positive rates, which can be explained by structural and systemic barriers that can go beyond individual motivation. The barrier analysis revealed lack of training as the most common barrier with more than two out of three non-users reporting insufficient training as one of the barriers to technology adoption. This implies the importance of educational preparedness as a factor in technology adoption. Most dental schools today offer minimal formal training in artificial intelligence, data science, or digital health technologies, producing a workforce that is conceptually fluent but virtually ill-equipped to work with these technologies in practice [17, 18]. The introduction of artificial intelligence literacy into undergraduate and postgraduate dental education courses, including supervised clinical exposure to artificial intelligence-aided diagnostic processes, is a necessary condition that can facilitate the rapid adoption. The barriers whose basis was mentioned by more than half of non-adopting respondents include cost-related factors to reflect the economic realities of dental practice, especially in the context of a private practice where decisions concerning capital investment must be supported by some form of demonstrated clinical or financial benefit. This barrier could be partially addressed by the creation of affordable and cloud-based artificial intelligence tools based on subscription models, which can decrease the required initial capital and allow them to be scaled [19]. Also, institutional support deficiencies are identified as a major hindrance, which implies that organizational culture and leadership dedication are also meaningful in supporting the adoption of technology in the dental environment. The ethical issues mentioned by 40 percent of the respondents are to be taken into account. Patient data privacy concerns, transparency of the algorithms, clinical responsibility of the artificial intelligence-aided decision-making and the possible replacement of the professional role are all valid and materially significant issues that have to be resolved with the help of effective ethical standards and clear regulations 20, 21]. Formulation of professional guidelines outlining the right extent of using artificial intelligence, defining the standards of using algorithms and explaining the liability frameworks would give practitioners the regulatory assurance to operate with such technologies with confidence.

The strong linkage between professional specialization and knowledge and practice adoption scores indicates that the closeness to the clinical area where artificial intelligence applications have the greatest immediate significance augments the interest in such technologies. The highest adoption rates were observed in periodontists, who face the clinical challenges that the artificial intelligence tools can solve in their day to day routine, which aligns with the hypothesis that perceived clinical relevance is an effective motivator of technology acceptance [22]. This result also contributes to the plan to implement the concept of artificial intelligence training into specialty-based clinical settings as opposed to providing it as isolated technical education. The cross-sectional research design does not allow it to make any causal conclusions about the determinants of the adoption of artificial intelligence. Self-reported information is prone to the social desirability effect, which can exaggerate the levels of positive attitudes and exaggerate the actual practice involvement. The methodology used in electronic distribution could have created selection bias in respondents who are technologically literate. Moreover, the research failed to determine the depth or quality of the artificial intelligence use in the individuals reporting active use and it also failed to determine the clinical impact of the artificial intelligence-assisted practice on patient outcomes. The study population could also be constrained by the geographic nature of the research and hence not be generalizable to a different context in terms of educational systems, regulatory arrangements and technological arrangements. Future studies ought to take the longitudinal designs to monitor the alterations of the knowledge; attitude and practice after specific educational interventions are implemented. Greater contextual insight of facilitators and barriers of adoption would be in the form of mixed-methods incorporating a qualitative exploration of the experiences of practitioners. Research studies that could explore the relationship between the use of artificial intelligence and the quantifiable clinical outcomes in periodontal practice would support the adoption recommendations. Also, the cross-country and health care system comparative studies would shed light on the contextual effects on the patterns of integrating artificial intelligence.

## Conclusion:

Dentists showed adequate awareness and positive attitudes toward artificial intelligence in periodontics; however, its clinical implementation remains limited. Key barriers include lack of training, cost constraints, infrastructural limitations and ethical concerns rather than attitudinal resistance. Addressing these gaps through structured education, affordable technologies and clear regulatory frameworks is essential for effective integration of artificial intelligence into periodontal practice.

## Figures and Tables

**Table 1:** Knowledge of artificial intelligence in periodontics among study participants (n = 800)

**Knowledge Domain**	**Correct responses (n)**	**Percentage (%)**
Familiarity with basic AI concepts	600	75
Knowledge of AI applications in periodontics	520	65
Awareness of AI-based periodontal tools	320	40
Understanding of AI limitations and errors	480	60

**Table 2:** Attitude and practice regarding artificial intelligence in periodontics among study participants (n = 800)

**Parameter**	**Positive Responses (n)**	**Percentage (%)**
Belief in AI reliability for dental applications	560	70
Willingness to adopt AI tools	640	80
Current use of AI in clinical practice	200	25
Perception of ethical concerns regarding AI	320	40

**Table 3:** Barriers to artificial intelligence adoption reported by non-users (n = 600)

**Barrier**	**Respondents (n)**	**Percentage (%)**
Insufficient training and education	410	68.3
High cost of AI technologies	325	54.2
Lack of institutional support and infrastructure	285	47.5
Concerns about accuracy and reliability	230	38.3
Ethical and medicolegal uncertainties	195	32.5
Resistance to change in clinical workflow	148	24.7
